# Effects of Thymol Supplementation in Health Care Sand on Growth Performance, Serum Biochemical Parameters, Fecal Microbial Diversity, and Fecal Metabolites in Squabs

**DOI:** 10.3390/ani16203314

**Published:** 2026-10-21

**Authors:** Chen Ma, Chao Li, Haiying Li, Jiajia Liu, Kunyu Liao, Yuanhao Li, Jie Ren, Guoqiang Wei, Xiaobin Li

**Affiliations:** 1 College of Animal Science, Xinjiang Agricultural University, Urumqi 830052, China; 2 Apiculture Technology Development Center of Xinjiang Uygur Autonomous Region, Urumqi 830001, China; 3 Institute of Animal Husbandry, Xinjiang Academy of Animal Husbandry, Urumqi 830011, China

**Keywords:** health care sand, thymol, squabs, antioxidant capacity, fecal metabolites

## Abstract

This study aimed to investigate the effects of thymol supplementation in health care sand on growth performance, serum biochemical parameters, fecal microbial diversity, and fecal metabolites in Grey King squabs. A total of 160 healthy 28-day-old Grey King squabs were selected and randomly assigned to four treatment groups: the CON, 2%Thy, 3%Thy, and 4%Thy groups. Each group consisted of 10 replicates with four squabs per replicate, resulting in a total of 40 squabs per group. Under identical feeding management conditions and dietary nutrient levels, squabs in the CON group received the basal diet plus 5 g of health care sand; those in the 2%Thy group received the basal diet plus 5 g of health care sand containing 2% thymol; those in the 3%Thy group received the basal diet plus 5 g of health care sand containing 3% thymol; and those in the 4%Thy group received the basal diet plus 5 g of health care sand containing 4% thymol. The experiment lasted for 30 days. During the experimental period, the pigeons were housed in cages measuring 45 cm × 50 cm × 60 cm. Each pigeon was provided with 40 g of diet and 5 g of health care sand daily and had free access to water. After Benjamini–Hochberg false discovery rate correction, no significant differences were observed in growth performance, body measurements, apparent nutrient retention, or energy metabolizability among treatments (*q* > 0.05). However, thymol-supplemented groups exhibited significantly increased serum total antioxidant capacity and superoxide dismutase, glutathione peroxidase, and catalase activities, together with decreased malondialdehyde levels (*q* < 0.05). Fecal microbiome and metabolomic analyses were restricted to the CON and 3%Thy groups, with the 3%Thy group selected after evaluation of the phenotypic and physiological responses rather than being prespecified before the experiment. These omics comparisons were therefore considered exploratory. No statistically significant difference in overall fecal microbial community composition was detected between the two groups, whereas 17 candidate differential fecal metabolite features were observed. These features were mainly putatively annotated as lipids and lipid-like molecules, organic acids and derivatives, organoheterocyclic compounds, and benzenoids. Among the supplementation levels evaluated, the 3%Thy group showed comparatively favorable numerical responses in several measured parameters and may be considered a candidate level for further investigation. However, the present findings do not establish an optimal thymol supplementation level, and the biological significance of the exploratory fecal omics results requires further validation.

## Simple Summary

Young pigeons experience physiological challenges during the transition from parental feeding to independent feeding, which may affect their growth and health. In this study, thymol, a plant-derived compound, was added at different levels to the health care sand provided to young pigeons. Thymol supplementation was associated with favorable changes in serum antioxidant parameters, whereas differences in growth performance and apparent nutrient utilization were not statistically significant after correction for multiple testing. Fecal microbiome and metabolomic analyses were conducted only in the control group and the 3% thymol group, which was selected after evaluation of the growth and physiological results. These exploratory analyses did not detect significant differences in overall fecal microbial community composition but identified several candidate differential fecal metabolite features. However, these findings do not establish that the observed microbial or metabolic associations caused the antioxidant responses or that 3% represents an optimal supplementation level. Further studies are needed to clarify the biological significance of these findings and determine an appropriate thymol supplementation level.

## 1. Introduction

Weaning is a critical transition in the growth and development of mammals, marking the shift from dependence on maternal nutrient provision to completely independent feed intake [1]. During this period, mammals are exposed to multiple concurrent stressors, including changes in feed form and environmental conditions as well as separation from the mother, making them highly susceptible to weaning stress [2]. Unlike mammals, most poultry species do not undergo a strictly defined weaning process. However, pigeons are altricial birds with a unique mode of parental rearing. After hatching, squabs are fed crop milk by their parent pigeons. At approximately 21–28 days of age, the parent pigeons gradually cease feeding, and the squabs transition to completely independent feed intake and enter the post-weaning stage [3]. At this stage, however, the digestive system of the weaned squabs is not yet fully developed, and their immune function remains relatively immature. Together with the abrupt transition from parental feeding to independent feed intake, these factors make them particularly susceptible to weaning stress [4]. Weaning stress can lead to reduced feed intake, impaired growth, compromised intestinal barrier function, decreased immune function, and aggravated oxidative stress and, in severe cases, may even result in mortality, thereby causing substantial economic losses to animal production [5]. Therefore, alleviating weaning stress in young animals and ensuring a smooth transition through the weaning period are of considerable importance. Current strategies for alleviating weaning stress in young animals mainly include optimizing feeding management, improving environmental conditions, adjusting dietary nutrient levels, and appropriately using feed additives [6]. Among these approaches, feed additives have become a major research focus because of their ease of application and marked effectiveness. In mammalian studies, Xu et al. [7] found that supplementation with carvacrol in weaned piglets (Large White × Landrace) alleviated intestinal damage caused by weaning stress, maintained intestinal barrier integrity, enhanced intestinal digestive enzyme activities, increased the expression of nutrient transport proteins, and improved feeding and digestive behavior in piglets. Li et al. [8] reported that thymol supplementation in heat-stressed lactating Murrah buffaloes significantly increased dry matter (DM) intake and ether extract (EE) digestibility, while significantly decreasing malondialdehyde (MDA) and cortisol (COR) levels and significantly increasing superoxide dismutase (SOD), glutathione peroxidase (GSH-PX), and catalase (CAT) activities; meanwhile, the composition of the rumen microbiota was also significantly altered. Carvacrol and thymol are monoterpenoid phenolic isomers derived from plants, both of which have been reported to improve animal growth performance and exert antibacterial, anti-inflammatory, and antioxidant effects, as well as to improve intestinal health [9]. In poultry studies, Liu et al. [10] demonstrated that dietary supplementation with 600 g/t of a thymol–carvacrol mixture significantly increased average body weight and average daily feed intake in broilers, increased jejunal villus height and the villus height-to-crypt depth ratio, and modulated the structure of the cecal microbiota. Yang et al. [11] also found that dietary supplementation with 60 mg/kg of a thymol–carvacrol cocrystal significantly increased daily weight gain and SOD activity in the serum and liver of broilers, reduced MDA levels, and increased cecal butyrate concentrations and the abundance of Lactobacillus, thereby improving antioxidant status and intestinal health. Based on the above background, weaning stress in squabs is a key issue affecting healthy pigeon production, whereas thymol has the potential to alleviate animal stress, improve intestinal health, and enhance immune function. Therefore, in the present study, 28-day-old Grey King pigeons were used to investigate the effects of different levels of thymol supplementation in health care sand on growth and development, nutrient digestion and metabolism, serum biochemical parameters, fecal microbial diversity, and fecal metabolites in squabs. The findings of this study are intended to provide a theoretical basis for the scientific application of thymol in the healthy production of squabs.

## 2. Materials and Methods

### 2.1. Animal Ethics Statement

All animal care and experimental procedures were conducted in accordance with the Guidelines for the Care and Use of Laboratory Animals in China and were approved by the Laboratory Animal Ethics Committee of Xinjiang Agricultural University (Approval No. 202602).

### 2.2. Experimental Materials

The thymol used in this study was purchased from Qingdao Mude Biotechnology Co., Ltd. (Qingdao, China) and had a thymol content of >90%.

### 2.3. Experimental Animals and Design

The animal experiment was conducted at Xinjiang Hetian Lanhai Pigeon Industry Development Co., Ltd. (Hetian, China). A single-factor experimental design was used. A total of 160 healthy 28-day-old Grey King squabs were randomly allocated to four treatment groups: the control group (CON), 2%Thy group, 3%Thy group, and 4%Thy group. Each group consisted of 10 replicates with four squabs per replicate, resulting in 40 squabs per group. Under identical feeding management and dietary nutrient conditions, squabs in the CON group received the basal diet plus 5 g of health care sand; those in the 2%Thy group received the basal diet plus 5 g of health care sand containing 2% thymol; those in the 3%Thy group received the basal diet plus 5 g of health care sand containing 3% thymol; and those in the 4%Thy group received the basal diet plus 5 g of health care sand containing 4% thymol. The experimental period lasted 30 days. Because information on thymol supplementation in health care sand for squabs is currently unavailable, the inclusion levels used in the present study were selected as an exploratory graded range rather than being derived by direct dose conversion from a previous study. Previous poultry studies using thymol or phenolic monoterpene-containing preparations were considered when defining the supplementation range, including the study of Mirderikvandi et al. [12]; however, differences in species, preparation, administration route, and exposure matrix preclude direct dose equivalence. Each squab was nominally allocated 5 g of health care sand daily. Based on this allocation, inclusion of the thymol-containing product at 2%, 3%, and 4% corresponded to theoretical product supplies of 100, 150, and 200 mg/bird/day, respectively. Given the stated thymol content of >90%, the corresponding theoretical amounts of thymol supplied were >90, >135, and >180 mg/bird/day for the 2%Thy, 3%Thy, and 4%Thy groups, respectively. These amounts represent theoretical supplies rather than measured individual intakes. The experimental pigeons were housed in cages measuring 45 cm × 50 cm × 60 cm. Each experimental replicate consisted of four squabs sharing a feeding trough, with 160 g of basal diet supplied per replicate daily, equivalent to a nominal allocation of 40 g per squab per day. Each squab was allocated 5 g of health care sand daily, and water was provided ad libitum. During the experimental period, routine visual observations revealed no obvious refusals of the basal diet or health care sand. However, feed and health care sand refusals and potential spillage were not quantitatively weighed, and individual consumption could not be verified. Therefore, actual feed and thymol intakes were not quantified, and feed conversion ratio (FCR) was not calculated. A 16-h daily photoperiod was maintained, and the ambient temperature was controlled at 15 ± 5 °C. Squabs were vaccinated according to the routine immunization program of the farm. The experiment was conducted in an open-sided pigeon house, with the same personnel responsible for animal management and experimental procedures throughout the study. The pigeon house was thoroughly washed and disinfected before the experiment, and routine disinfection was subsequently performed according to standard farm practices. The composition and nutrient levels of the basal diet are presented in Table 1, and the composition of the health care sand is presented in Table 2. All ingredients used in the health care sand formulations, except thymol, were supplied by Xinjiang Hetian Lanhai Pigeon Industry Development Co., Ltd. The herbal preparations and other functional supplements used across the four treatment groups were obtained from the same respective products and production batches. The formulations were adapted from the health care sand formulation routinely used at the farm. To accommodate increasing levels of thymol, the proportions of medium sand, red clay, and limestone powder were deliberately reduced, while the inclusion percentages of all other ingredients were maintained unchanged. Medium sand primarily served as a granular carrier, red clay contributed to the physical properties of the mixture, and limestone powder provided a source of calcium.

### 2.4. Measurements and Methods

#### 2.4.1. Growth Performance Data Collection

Fasting body weight of the squabs was measured on days 0 and 30 of the experiment using an electronic balance (maximum weighing capacity: 1 kg; Shanghai Yueping Instrument Co., Ltd., Shanghai, China). During the experimental period, each replicate of four squabs was supplied with 160 g of basal diet per day through a shared feeding trough, corresponding to a nominal allocation of 40 g per bird. Health care sand was supplied at a nominal allowance of 5 g per bird daily. Routine visual observations revealed no obvious refusals of either the diet or health care sand. However, refusals and potential spillage were not quantitatively weighed, and individual consumption could not be verified. Therefore, actual feed intake was not determined, and feed conversion ratio (FCR) was not calculated. The average body weight, total weight gain, and average daily gain (ADG) of squabs under the different health care sand treatments were subsequently calculated.

#### 2.4.2. Body Measurement Parameters

Body measurements of the squabs were taken on days 0 and 30 of the experiment. Breast width, breast depth, shank length, keel length, oblique body length, and shank circumference were measured using a flexible measuring tape and a digital vernier caliper (measuring range: 0–150 mm; accuracy: 0.02 mm). The digital vernier caliper was supplied by Harbin Measuring & Cutting Tool Group Co., Ltd., Harbin, China. The total increase in each morphological measurement from day 0 to day 30 of the experiment was subsequently calculated. All measurements were performed according to the standard terminology and measurement methods for poultry production performance.

#### 2.4.3. Apparent Nutrient Retention and Energy Metabolizability

The total excreta collection method was used to evaluate apparent nutrient retention and energy metabolizability in squabs. From days 28 to 30 of the experiment, one healthy squab was randomly selected from each replicate and individually housed in a metabolic cage for the digestibility trial, resulting in a total of 10 samples per treatment group for subsequent analysis. Before formal sample collection, the squabs were fasted for 8 h to clear the intestinal contents. Thereafter, each squab was provided with 40 g of diet and 5 g of experimental health care sand daily for three consecutive days. All excreta produced during the 3-day collection period were collected separately from each cage and used as representative samples. Feed and air-dried excreta samples were oven-dried at 65 °C to constant weight, ground, and passed through a fine-mesh sieve for chemical analysis. Before oven drying, the excreta samples were first air-dried to prevent crust formation, minimize the loss of volatile compounds, and inhibit microbial activity, thereby ensuring the accuracy of subsequent chemical analyses.

The concentrations of dry matter (DM), organic matter (OM), crude protein (CP), ether extract (EE), and gross energy (GE) were determined in the diet and collected excreta. Apparent nutrient retention and energy metabolizability were subsequently calculated from the corresponding nutrient or energy intake and total excreta output during the collection period. All analyses were conducted in accordance with the relevant Chinese National Standards: DM, GB/T 6435-2014 [13]; OM, GB/T 6438-2007 [14]; GE, GB/T 45104-2024 [15]; CP, GB/T 6432-1994 [16]; and EE, GB/T 6433-2006 [17]. Apparent metabolizable energy (AME) was calculated using the following equation: AME (kcal/kg DM) = (GE intake − GE excretion)/dry matter intake.

#### 2.4.4. Serum Parameters

At the end of the 30-day trial, one squab was randomly chosen from each replicate after 4 h of fasting (10 birds per treatment). Blood (3 mL per bird) was drawn from the wing vein into tubes without anticoagulant. Serum was separated by centrifugation at 3500 r/min for 10 min using a TDL-5-A centrifuge (Shanghai Anke Instrument Co., Ltd., Shanghai, China), transferred to 1.5 mL cryogenic tubes, and stored at −80 °C until analysis. Serum biochemical and antioxidant measurements were performed by Beijing Huaying Biotechnology Co., Ltd. (Beijing, China).

The measured parameters were divided into two categories: (1) serum biochemical parameters, including total cholesterol (TC), triglycerides (TG), total protein (TP), albumin (ALB), globulin (GLB), uric acid (Ua), glucose (GLU), acid phosphatase (ACP), and alkaline phosphatase (ALP), and (2) serum oxidative and antioxidant parameters, including total antioxidant capacity (T-AOC), superoxide dismutase (SOD), glutathione peroxidase (GSH-PX), catalase (CAT) activity, and malondialdehyde (MDA) content.

#### 2.4.5. Fecal Microbial Diversity Analysis

Bacterial genomic DNA was isolated from 20 fecal samples, comprising 10 samples each from the CON and 3%Thy groups, using the E.Z.N.A. Soil DNA Kit (Omega Bio-tek, Norcross, GA, USA) according to the manufacturer’s protocol. DNA concentration and purity were assessed with a NanoDrop 2000 spectrophotometer (Thermo Fisher Scientific, Wilmington, DC, USA), while integrity was examined by electrophoresis on a 1% agarose gel. Samples meeting the quality requirements were stored at −80 °C until amplification. The V3–V4 region of the bacterial 16S rRNA gene was amplified with primers 338F (5′-ACTCCTACGGGAGGCAGCAG-3′) and 806R (5′-GGACTACHVGGGTWTCTAAT-3′). Each 20-μL reaction contained 10 ng of template DNA, 4 μL of 5× FastPfu buffer, 2 μL of 2.5 mM dNTPs, 0.8 μL of each primer (5 μM), 0.4 μL of FastPfu DNA polymerase (TransGen Biotech, Beijing, China), and sterile deionized water. Amplification began with denaturation at 95 °C for 3 min, followed by 27 cycles of 95 °C for 30 s, 55 °C for 30 s, and 72 °C for 45 s. A final extension was performed at 72 °C for 10 min. Amplicons were examined on a 2% agarose gel, purified with the AxyPrep DNA Gel Extraction Kit (Corning Incorporated, Union City, CA, USA), and quantified using the QuantiFluor-ST system. Purified amplicons were then pooled at equimolar concentrations and sequenced on an Illumina MiSeq platform using paired-end 300-bp reads. Sequencing was conducted by Novogene Co., Ltd. (Beijing, China).

Raw sequencing reads were first demultiplexed according to sample-specific barcodes and quality-filtered using fastp (version 0.23.4) to remove adapter sequences and low-quality reads. The processed sequences were subsequently analyzed using QIIME 2 (version 2020.2), and further quality filtering, denoising, paired-end read merging, and chimera removal were performed using the q2-dada2 plugin (version 2020.2.0), thereby generating high-resolution amplicon sequence variants (ASVs). Compared with the conventional operational taxonomic unit (OTU)-based clustering approach, the ASV-based approach provides higher taxonomic resolution at the single-nucleotide level.

Representative ASV sequences were taxonomically classified using a naive Bayes classifier against the SILVA 16S rRNA reference database (release 138). To minimize potential bias arising from unequal sequencing depths among samples, the ASV table was rarefied before diversity analysis. A rarefaction depth of 77,904 sequences per sample was selected because this depth corresponded to the minimum number of denoised sequences among the 18 samples, thereby standardizing sequencing effort while retaining all samples for downstream analysis. At this sequencing depth, the rarefaction curves approached a plateau, indicating adequate coverage of the dominant microbial communities. However, because rarefaction may reduce the detection of low-abundance ASVs, richness-related results were interpreted with appropriate caution. Alpha diversity indices, including Chao1, dominance, goods_coverage, observed_features, pielou_e, Shannon, and Simpson indices, were subsequently calculated in QIIME 2 to evaluate microbial richness and diversity within samples. Beta diversity was visualized using principal component analysis (PCA) and principal coordinates analysis (PCoA). PCA was used to reduce the dimensionality of the microbial abundance data by decomposing the variance in the multivariate dataset, with Euclidean distances underlying the ordination. In addition, PCoA was performed based on an unweighted UniFrac distance matrix to visualize phylogeny-based differences in microbial community composition between the CON and 3%Thy groups. Differences in overall fecal microbial community composition between the two groups were further evaluated using permutational multivariate analysis of variance (PERMANOVA; Adonis) based on the unweighted UniFrac distance matrix, with 999 permutations. For microbiome analyses, Benjamini–Hochberg false discovery rate (FDR) correction was performed separately within each family of tests, including taxa at the same taxonomic level or pairwise comparisons of the same diversity index. The FDR-adjusted *p* values are hereafter referred to as q values, and q < 0.05 was considered statistically significant, where applicable. Linear discriminant analysis effect size (LEfSe) was used as an exploratory approach to screen for candidate discriminatory taxa between the CON and 3%Thy groups. Taxa meeting the specified statistical screening threshold and an LDA score > 2.0 were considered candidate discriminatory taxa rather than confirmed treatment-responsive biomarkers. Functional prediction was performed using Tax4Fun2 (version 1.1.5) based on SILVA release 138 annotation information. Microbiome-related analyses and visualization were performed using QIIME 2, R software (version 4.3.1), and the ggplot2 package (version 3.5.1).

#### 2.4.6. Fecal Metabolite Analysis

For metabolite extraction, approximately 20 ± 5 mg of fecal material was mixed with a 6 mm grinding bead and 0.4 mL of methanol–water (4:1, *v*/*v*) containing 0.02 mg/mL L-2-chlorophenylalanine as an internal standard. Samples were homogenized at 50 Hz for 6 min at −10 °C, followed by sonication at 5 °C for 30 min. After incubation at −20 °C for 30 min to promote protein precipitation, the extracts were centrifuged at 13,000× *g* for 15 min at 4 °C. The resulting supernatants were transferred to sample vials for LC-MS/MS analysis. To monitor the stability and reproducibility of the analytical system, quality control (QC) samples were prepared by pooling equal aliquots of all experimental samples. One QC sample was injected after every 10 experimental samples throughout the analytical sequence. Metabolomic analysis of the fecal samples was performed by Shanghai Majorbio Bio-Pharm Technology Co., Ltd. (Shanghai, China) using an ultra-high-performance liquid chromatography–tandem mass spectrometry (UHPLC-MS/MS) platform consisting of an ACQUITY UPLC system (Waters Corporation, Milford, MA, USA) coupled to an AB SCIEX TripleTOF 6600 high-resolution mass spectrometer (AB Sciex Pte. Ltd., Singapore). The mass spectrometer was equipped with an electrospray ionization (ESI) source and operated in both positive- and negative-ion modes. Data were acquired in data-dependent acquisition (DDA) mode over a full-scan mass range of *m*/*z* 50–1200. Raw LC-MS data were preprocessed using Progenesis QI software (version 3.0; Waters Corporation, Milford, MA, USA), including peak detection, retention-time alignment, and feature extraction. Internal standard peaks and known false-positive signals, including background noise, column bleed, and derivatization reagent-related peaks, were removed from the data matrix, followed by data redundancy reduction and peak merging. Metabolite features were annotated by comparing accurate precursor mass measurements and MS/MS fragmentation information with entries in the Human Metabolome Database (HMDB), METLIN, and the Majorbio database. The mass-error tolerance was set to less than 10 ppm. According to the annotation confidence assessment provided by the analytical laboratory, both Level B(i) and Level B(ii) annotations were classified as MSI Level 2 (putatively annotated compounds). Level B(i) annotations were supported by experimental MS/MS spectral library matching, whereas Level B(ii) annotations were based on theoretically predicted fragmentation information. Experimental MS/MS spectra were compared with reference spectral information where available. Although authentic reference standards were used in the analytical workflow, not all annotated metabolites were individually confirmed using corresponding standards. Therefore, these metabolite assignments were treated as putative annotations. Blank samples were included in the laboratory’s analytical quality control procedures to assess potential background contamination. Pooled QC samples were used to monitor analytical reproducibility. Background contamination assessment and metabolite identification confidence were considered distinct aspects of data quality evaluation.

The obtained metabolite abundance matrix was subjected to missing-value filtering and normalization, followed by data transformation and scaling as required for subsequent analyses. Principal component analysis (PCA) was used as an unsupervised method to characterize the overall metabolic profiles of the samples and the distribution trends between groups. Partial least squares discriminant analysis (PLS-DA) was subsequently performed to further evaluate differences in metabolic profiles between the CON and 3%Thy groups, and permutation testing was used to assess the stability of the PLS-DA model and potential overfitting. Variable importance in projection (VIP) values were calculated based on the orthogonal partial least squares discriminant analysis (OPLS-DA) model and, together with univariate statistical analysis, were used to identify differential metabolites between the CON and 3%Thy groups. Differential metabolites were screened using the criteria of VIP > 1 and q < 0.05. Volcano plots were used to visualize the direction and magnitude of changes in differential metabolites, and differential metabolites with relatively high VIP values were subjected to abundance variation and clustering analyses. Identified metabolites were classified at the Superclass level according to chemical classification information available in databases such as the Human Metabolome Database (HMDB). Differential metabolites were mapped to the Kyoto Encyclopedia of Genes and Genomes (KEGG) database for functional annotation and metabolic pathway enrichment analysis. False discovery rate (FDR) correction was performed using the Benjamini–Hochberg method, and q < 0.05 was considered the threshold for significant enrichment.

#### 2.4.7. Correlation Analysis

Spearman rank correlation analysis was performed using GraphPad Prism software (version 10.0.0; GraphPad Software Inc., San Diego, CA, USA) to evaluate the correlations between the differential bacterial taxa identified by LEfSe and the top 20 metabolites ranked by VIP. To avoid duplication within the same taxonomic lineage, only differential taxa at the genus level were included in the analysis. Correlations between the top 20 metabolites ranked by VIP and the five antioxidant parameters were also analyzed. Benjamini–Hochberg false discovery rate (FDR) correction was applied to all 180 correlation tests, and q < 0.05 was considered statistically significant. In the present study, body weight data were collected from all 160 squabs, whereas body measurement data were collected from two squabs randomly selected from each replicate within each treatment. For growth performance and body measurement parameters, the replicate was considered the experimental unit for statistical analysis. Although body weight was measured in all squabs, body weight-related parameters for the four squabs within each replicate were averaged, and the replicate mean was used as a single statistical observation. Therefore, each treatment had 10 statistical replicates (n = 10). Similarly, for body measurement parameters, the measurements obtained from the two selected squabs within each replicate were first averaged, and this mean value was used as the statistical observation for that replicate (n = 10). Considering experimental feasibility and analytical costs, before data analysis and after completion of the feeding trial, one squab was randomly selected from each replicate, resulting in 10 squabs per treatment for analysis of serum biochemical and antioxidant parameters. The same 10 animals within each treatment were used for both serum biochemical and antioxidant analyses. Because only one squab was randomly sampled from each replicate, each treatment included 10 biological replicates for these analyses (n = 10). After completion of the feeding trial and evaluation of the growth performance, body measurements, and serum biochemical and antioxidant responses across the four treatment groups, the CON and 3%Thy groups were selected for subsequent fecal microbiome and metabolomic analyses because the 3%Thy group exhibited the most consistent favorable phenotypic and physiological responses among the thymol-supplemented groups. This selection was made after evaluation of the treatment responses and was not prespecified before the experiment. Therefore, the subsequent microbiome and metabolomic comparisons between the CON and 3%Thy groups were considered secondary exploratory analyses rather than confirmatory analyses of a dose-dependent thymol effect. One sample was randomly selected from each replicate in the CON and 3%Thy groups, resulting in 10 fecal samples per treatment for fecal microbiota and metabolite analyses. During the fecal microbiota analysis, one sample from the CON group exhibited unusually high microbial richness, with Chao1 and observed_features values more than sixfold higher than those of the other CON samples. This sample was excluded from the original analysis based on a post hoc assessment rather than a prespecified quality-control criterion. Subsequently, one sample from the 3%Thy group was randomly excluded solely to obtain equal sample sizes for the original between-group comparisons. This additional exclusion was not based on sequencing quality or an abnormal microbial profile. Equal group sizes were not required for the statistical analyses performed in this study. Consequently, nine fecal samples per group were included in the reported microbiome analysis (n = 9 per group), whereas all 10 fecal samples per group were retained for the metabolomic analysis (n = 10 per group). No mortality or apparent health abnormalities occurred during the experimental period. The sample sizes were determined with reference to common practice in fecal microbiome and metabolomics studies, balancing analytical robustness with sequencing and analytical costs.

### 2.5. Statistical Analysis

Growth performance, apparent nutrient retention, energy metabolizability, serum biochemical parameters, and serum antioxidant parameters are presented as means ± standard deviations (SDs), whereas quantitative fecal microbiome and metabolomic data are presented as means ± standard errors of the mean (SEMs), where applicable. Conventional statistical analyses were performed using SPSS software (version 27.0). No single primary outcome was prespecified before the experiment because the study was designed to evaluate responses across multiple physiological domains. For multiplicity control, conventional treatment-response endpoints were grouped according to their biological domains: (1) growth and morphometric responses, (2) apparent nutrient retention and energy metabolizability, (3) serum biochemical parameters, and (4) antioxidant parameters. For growth and morphometric analyses, total weight gain and changes from day 0 to day 30 were treated as treatment-response endpoints, whereas the raw day-0 and day-30 measurements were retained descriptively. Because average daily gain (ADG) was mathematically derived from total weight gain over the fixed 30-day experimental period, it was not treated as an independent endpoint in the FDR adjustment. Before statistical analysis, data normality was assessed using the Shapiro–Wilk test and homogeneity of variances using Levene’s test. When homogeneity of variance was satisfied, comparisons among the four treatment groups (CON, 2%Thy, 3%Thy, and 4%Thy) were performed using one-way analysis of variance (ANOVA); when variance heterogeneity was detected, Welch’s ANOVA was used. The Benjamini–Hochberg false discovery rate (FDR) procedure was applied to the omnibus *p* values within each biological domain, and *q* < 0.05 was considered statistically significant. For endpoints retaining a significant overall treatment effect after FDR correction, Tukey’s multiple-comparison test was used for pairwise comparisons when variances were homogeneous, whereas the Games–Howell test was used following Welch’s ANOVA when variances were heterogeneous. For other parametric comparisons between the CON and 3%Thy groups, a two-tailed independent-samples Student’s *t*-test was used. Other statistical analyses of the fecal microbiome and untargeted metabolomics data were performed according to the corresponding omics analysis methods.

## 3. Results

### 3.1. Effects of Thymol Supplementation in Health Care Sand on Growth Performance and Body Measurement Parameters of Squabs

The effects of thymol supplementation in health care sand on body weight and body measurement parameters in squabs are presented in Table 3. After Benjamini–Hochberg FDR correction within the body weight and morphometric outcome domain, none of these variables differed significantly between the control and treatment groups or among the treatment groups (*q* > 0.05).

### 3.2. Effects of Thymol Supplementation in Health Care Sand on Apparent Nutrient Retention and Energy Metabolizability in Squabs

The effects of thymol supplementation in health care sand on apparent nutrient retention and energy metabolizability in squabs are presented in Table 4. After Benjamini–Hochberg false discovery rate (FDR) correction within the apparent nutrient retention and energy metabolizability outcome domain, no statistically significant treatment effects were detected for any of the measured parameters (*q* > 0.05).

### 3.3. Effects of Thymol Supplementation in Health Care Sand on Serum Biochemical and Antioxidant Parameters in Squabs

**Figure 1:**
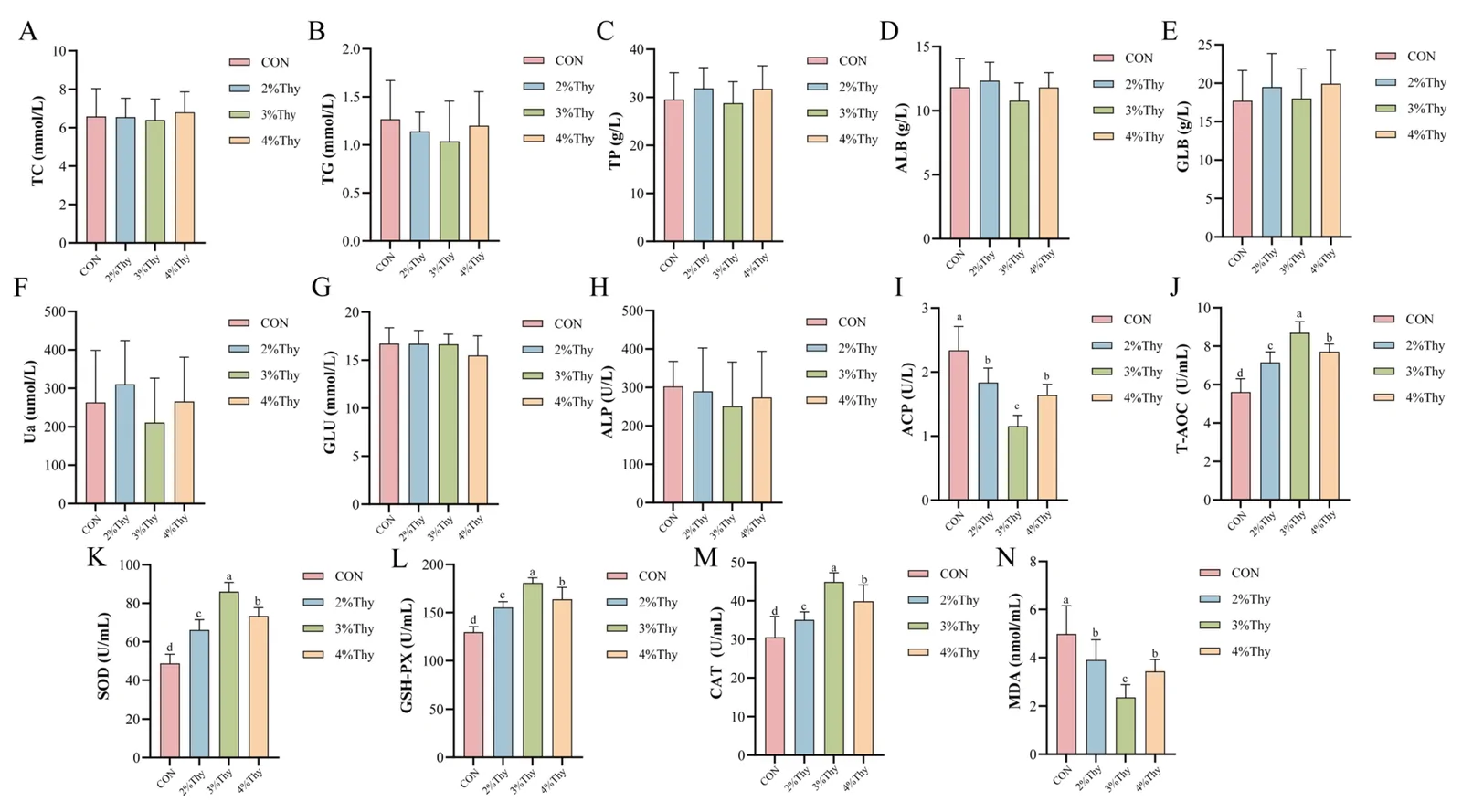
Effects of thymol supplementation in health care sand on serum biochemical and antioxidant parameters in squabs: (**A**) total cholesterol; (**B**) triglycerides; (**C**) total protein; (**D**) albumin; (**E**) globulin; (**F**) uric acid; (**G**) glucose; (**H**) alkaline phosphatase; (**I**) acid phosphatase; (**J**) total antioxidant capacity; (**K**) superoxide dismutase; (**L**) glutathione peroxidase. (**M**) catalase; (**N**) malondialdehyde. Data are presented as mean ± SD (n = 10 biological replicates per treatment). Overall treatment effects were evaluated using FDR-adjusted omnibus tests (q < 0.05). For outcomes with significant omnibus effects, different superscript letters indicate significant pairwise differences determined using Tukey’s or Games–Howell post hoc tests, as appropriate (adjusted *p* < 0.05).

The effects of thymol supplementation in health care sand on serum biochemical and antioxidant parameters in squabs are presented in Figure 1. Regarding serum biochemical parameters, after Benjamini–Hochberg FDR correction within the serum biochemical outcome domain, no significant differences were observed in TC, TG, TP, ALB, GLB, Ua, GLU, or ALP between the CON and treatment groups or among the treatment groups (*q* > 0.05) (Figure 1A–H and Table S1). In contrast, ACP differed significantly among treatments after FDR correction (*q* < 0.05). Compared with the CON group, ACP was significantly decreased in the 2%Thy, 3%Thy, and 4%Thy groups, and the 3%Thy group was also significantly lower than the 2%Thy and 4%Thy groups (*q* < 0.05), whereas no significant difference was observed between the 2%Thy and 4%Thy groups (*q* > 0.05) (Figure 1I). Regarding antioxidant parameters, all five antioxidant indicators showed significant treatment effects after Benjamini–Hochberg FDR correction within the antioxidant outcome domain (*q* < 0.05) (Table S2). Compared with the CON group, T-AOC, SOD, GSH-PX, and CAT were significantly increased in the thymol-supplemented groups, whereas MDA was significantly decreased (*q* < 0.05) (Figure 1J–N). Significant differences were also observed among some thymol-supplemented groups, as indicated by the different superscript letters in Figure 1 (*q* < 0.05).

### 3.4. Effects of Thymol Supplementation in Health Care Sand on Fecal Microbial Diversity in Squabs

**Figure 2:**
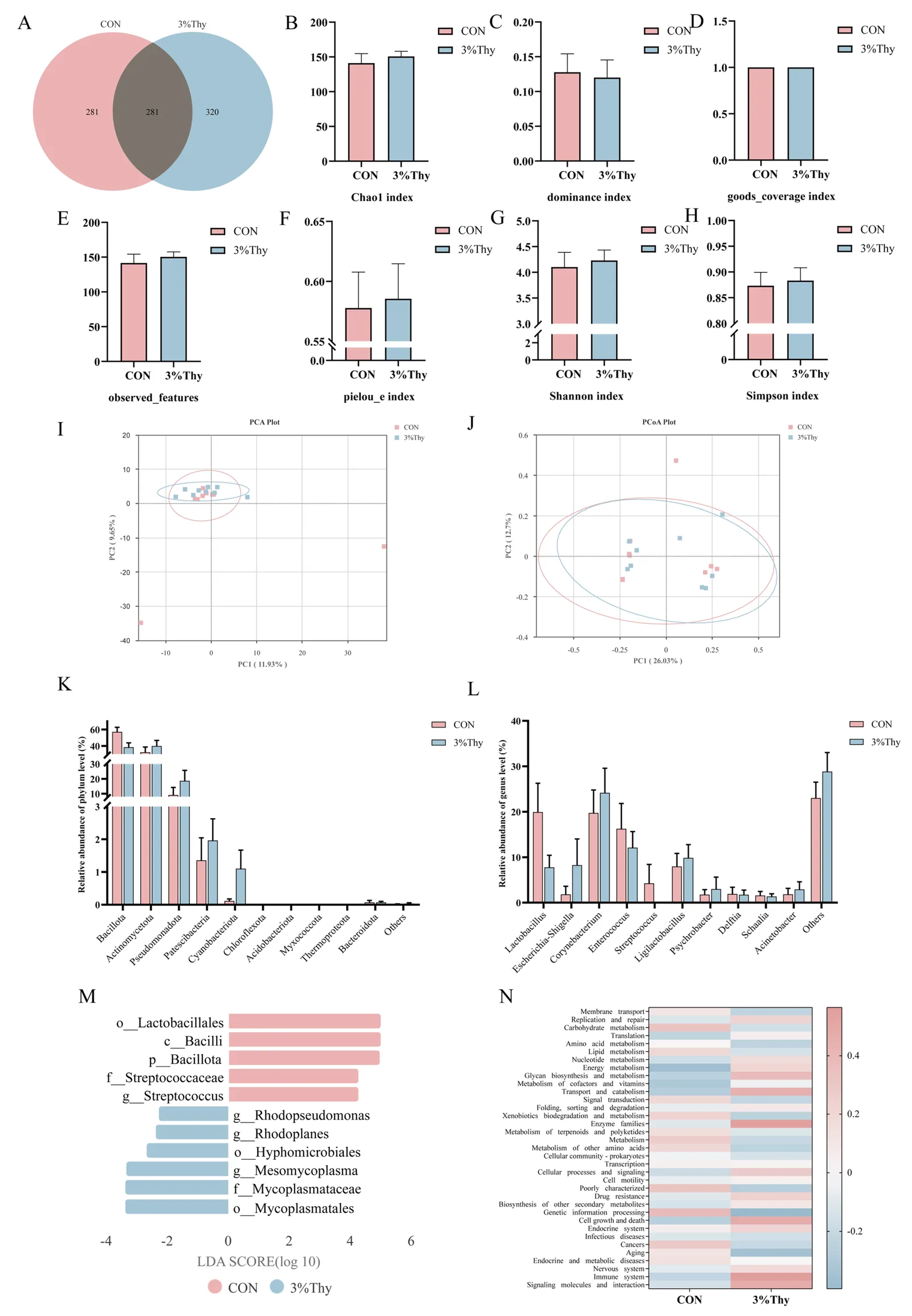
Effects of thymol supplementation in health care sand on fecal microbial diversity in squabs: (**A**) ASV-based Venn diagram analysis; (**B**) Chao1 index; (**C**) dominance index; (**D**) goods_coverage index; (**E**) observed_features index; (**F**) pielou_e index; (**G**) Shannon index; (**H**) Simpson index; (**I**) PCA based on Euclidean distances; (**J**) PCoA based on unweighted UniFrac distances; (**K**) effects of thymol supplementation in health care sand on the relative abundance of the fecal microbiota at the phylum level in squabs; (**L**) effects of thymol supplementation in health care sand on the relative abundance of the fecal microbiota at the genus level in squabs; (**M**) candidate discriminatory microbial taxa identified by exploratory LEfSe analysis (LDA score > 2.0); and (**N**) functional prediction of the microbiota based on Tax4Fun2. The CON and 3%Thy groups each included nine biological replicates. None of the functions predicted by Tax4Fun2 reached statistical significance after Benjamini–Hochberg false discovery rate (FDR) correction (*q* > 0.05). n = 9 per group. Values with no superscript letters or with the same superscript letters are not significantly different (*q* > 0.05), whereas values with different superscript letters differ significantly (*q* < 0.05).

The effects of thymol supplementation in health care sand on fecal microbial diversity in squabs are presented in Figure 2. A total of 882 ASVs were detected across all fecal samples, of which 281 ASVs were shared between the CON and 3%Thy groups, 281 ASVs were unique to the CON group, and 320 ASVs were unique to the 3%Thy group (Figure 2A). Based on the α-diversity analysis, no significant differences were observed between the CON and 3%Thy groups in the Chao1, dominance, goods_coverage, observed_features, pielou_e, Shannon, or Simpson indices (Figure 2B–H) (Table S3). PCA based on the Euclidean distance structure of the microbial abundance data showed partial overlap between samples from the CON and 3%Thy groups (Figure 2I). Similarly, PCoA based on the unweighted UniFrac distance matrix showed substantial overlap between the two groups (Figure 2J). To further evaluate differences in overall fecal microbial community composition, PERMANOVA was performed using the unweighted UniFrac distance matrix. No statistically significant difference was detected between the CON and 3%Thy groups (pseudo-F = 0.8166, R^2^ = 0.0486, *p* = 0.679; 999 permutations).

At the phylum level, the 10 most abundant phyla in pigeon feces were Bacillota, Actinomycetota, Pseudomonadota, Patescibacteria, Cyanobacteria, Bacteroidota, Chlamydiota, Bdellovibrionota, Chloroflexota, and Myxococcota (Figure 2K and Table S4). At the genus level, the 10 most abundant genera were Lactobacillus, Escherichia–Shigella, Corynebacterium, Enterococcus, Streptococcus, Ligilactobacillus, Psychrobacter, Delftia, Acinetobacter, and Limosilactobacillus (Figure 2L and Table S5). During the analysis of relative taxonomic abundance at the phylum and genus levels, false discovery rate (FDR) correction was applied to the *p* values to reduce the risk of false-positive findings, yielding FDR-adjusted *p* values (*q* values). The results showed no significant differences between the CON and 3%Thy groups at either the phylum or genus level (*q* > 0.05).

LEfSe analysis was conducted as an exploratory screening approach to identify potential taxonomic differences between the CON and 3%Thy groups. As shown in Figure 2M, 11 taxa met the LEfSe screening criteria. Five candidate taxa were associated with the CON group, including Bacillota, Bacilli, Lactobacillales, Streptococcaceae, and Streptococcus. Six candidate taxa were associated with the 3%Thy group, including Hyphomicrobiales, Mycoplasmatales, Mycoplasmataceae, Rhodopseudomonas, Rhodoplanes, and Mesomycoplasma. However, conventional relative abundance comparisons at the phylum and genus levels did not reveal significant differences after FDR correction (q > 0.05). Accordingly, these LEfSe results were regarded as exploratory candidate discriminatory taxa rather than confirmed treatment-related changes in microbial abundance. Functional prediction using Tax4Fun2 showed that, among the top 35 predicted functions, the CON group was mainly associated with carbohydrate metabolism, poorly characterized genetic information processing, and cancers, whereas the 3%Thy group was mainly associated with glycan biosynthesis and metabolism, transport and catabolism, enzyme families, cell growth and death, the immune system, and signaling molecules and interaction. However, after false discovery rate (FDR) correction of the raw *p* values, no significant differences were observed between the CON and 3%Thy groups (*q* > 0.05). Therefore, these predicted functional differences should be interpreted as exploratory findings rather than statistically significant functional alterations.

### 3.5. Effects of Thymol Supplementation in Health Care Sand on Fecal Metabolites in Squabs

**Figure 3:**
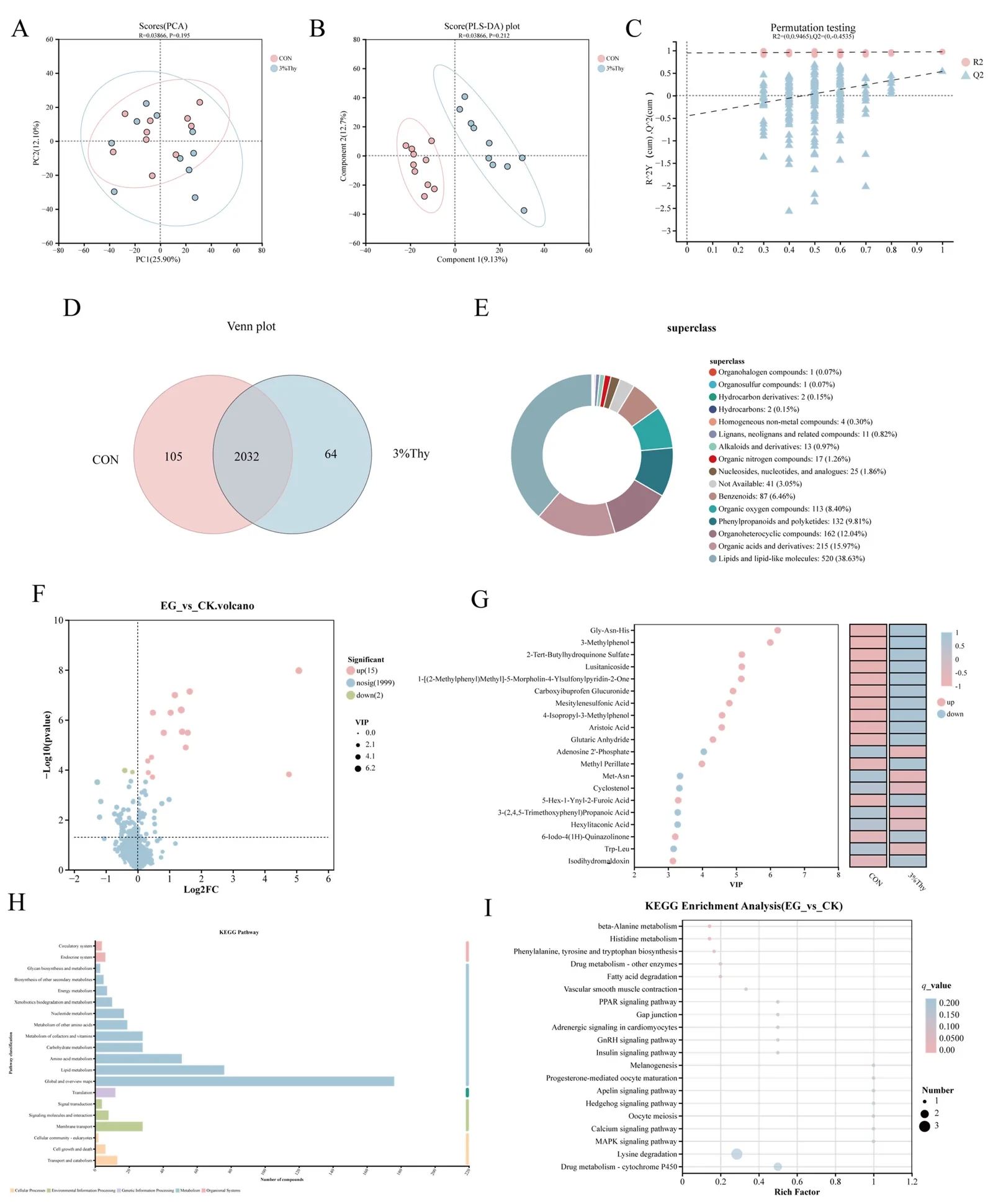
Effects of thymol supplementation in health care sand on the fecal metabolome of squabs: (**A**) principal component analysis (PCA); (**B**) partial least squares discriminant analysis (PLS-DA); (**C**) permutation test of the PLS-DA model; (**D**) Venn diagram of metabolites in the CON and 3%Thy groups; (**E**) Superclass classification of identified metabolites; (**F**) volcano plot of differential metabolites between the CON and 3%Thy groups; (**G**) top 20 differential metabolites ranked by VIP value; (**H**) KEGG pathway classification of differential metabolites; and (**I**) KEGG pathway enrichment analysis of differential metabolites. n = 10 per group.

To explore variation in the overall fecal metabolic profiles between the CON and 3%Thy groups, PCA was first performed (Figure 3A). The two groups showed partial separation with some overlap in the PCA score plot. PLS-DA was subsequently performed as an exploratory supervised analysis (Figure 3B), and the resulting score plot showed separation between the two groups. However, because PLS-DA uses group membership to construct the discriminant model, visual separation alone cannot establish a robust treatment-associated difference in the overall metabolome. A 200-iteration permutation test was conducted to assess potential overfitting (Figure 3C). The reported R^2^ and Q^2^ intercepts were 0.8236 and −0.2242, respectively. Although the negative Q^2^ intercept was consistent with reduced predictive performance after permutation of group labels, the overall reliability of the supervised model should be interpreted in conjunction with its original cross-validated performance parameters and the complete permutation-test results. Given the relatively small sample size (n = 10 per group), the supervised multivariate findings were regarded as exploratory rather than confirmatory.

Venn analysis was performed on the metabolites detected in the CON and 3%Thy groups (Figure 3D). A total of 2201 metabolites were detected across the two groups, of which 2032 were shared between the CON and 3%Thy groups, 105 were unique to the CON group, and 64 were unique to the 3%Thy group. These results indicate a high degree of similarity in the overall metabolite composition between the two groups, although the 3%Thy treatment still induced a certain degree of alteration in metabolite composition. Based on their chemical structures, the 2016 identified metabolites were classified, of which 1346 had Superclass classification information and were assigned to 16 categories (Figure 3E). These included 520 metabolites classified as Lipids and lipid-like molecules, accounting for 38.63%; 215 as Organic acids and derivatives, accounting for 15.97%; 162 as Organoheterocyclic compounds, accounting for 12.04%; 132 as Phenylpropanoids and polyketides, accounting for 9.81%; 113 as Organic oxygen compounds, accounting for 8.40%; 87 as Benzenoids, accounting for 6.46%; 41 as Not Available, accounting for 3.05%; 25 as Nucleosides, nucleotides, and analogues, accounting for 1.86%; 17 as Organic nitrogen compounds, accounting for 1.26%; 13 as Alkaloids and derivatives, accounting for 0.97%; 11 as Lignans, neolignans, and related compounds, accounting for 0.82%; 4 as Homogeneous non-metal compounds, accounting for 0.30%; 2 as Hydrocarbons, accounting for 0.15%; 2 as Hydrocarbon derivatives, accounting for 0.15%; 1 as Organosulfur compounds, accounting for 0.07%; and 1 as Organohalogen compounds, accounting for 0.07%. To further identify differential metabolites induced by the 3%Thy treatment, metabolites were screened using the combined criteria of VIP > 1 and q < 0.05 (Figure 3F). A total of 17 differential metabolites were identified between the CON and 3%Thy groups, of which 15 showed increased abundance and 2 showed decreased abundance, indicating that the 3%Thy treatment altered the abundance of multiple metabolites. Among the 17 differential metabolite annotations, 12 were classified as Level B(i) and five as Level B(ii) according to the Majorbio annotation system. Both categories were reported by the analytical laboratory as MSI Level 2 (putatively annotated compounds).

VIP values for the differential metabolites were calculated based on the OPLS-DA model, and metabolites with relatively high contributions were ranked accordingly (Figure 3G). The top 20 metabolites were Gly-Asn-His, 3-Methylphenol, 2-Tert-Butylhydroquinone Sulfate, Lusitanicoside, 1-[(2-Methylphenyl)Methyl]-5-Morpholin-4-Ylsulfonylpyridin-2-One, Carboxyibuprofen Glucuronide, Mesitylenesulfonic Acid, 4-Isopropyl-3-Methylphenol, Aristoic Acid, Glutaric Anhydride, Adenosine 2′-Phosphate, Methyl Perillate, Met-Asn, Cyclostenol, 5-Hex-1-Ynyl-2-Furoic Acid, 3-(2,4,5-Trimethoxyphenyl)Propanoic Acid, Hexylitaconic Acid, 6-Iodo-4(1H)-Quinazolinone, Trp-Leu, and Isodihydromaldoxin. These metabolites exhibited different patterns of relative abundance between the CON and 3%Thy groups. The clustering heatmap further showed that the abundances of some metabolites were markedly increased following the 3%Thy treatment, whereas those of others were markedly decreased, suggesting that these metabolites may be important contributors to the differences in metabolic profiles between the two groups. KEGG functional annotation indicated that the differential fecal metabolites were mapped to several database-defined biological pathways, including those related to amino acid metabolism, lipid metabolism, carbohydrate metabolism, and signal transduction (Figure 3H). KEGG pathway enrichment analysis further identified associations with pathways such as amino acid metabolism, fatty acid degradation, PPAR signaling, cGMP-PKG signaling, insulin signaling, and MAPK signaling (Figure 3I). These findings represent database-derived associations between the identified fecal metabolites and annotated pathways. They do not demonstrate that these pathways were activated, inhibited, or otherwise functionally altered in the tissues of squabs following thymol supplementation. Therefore, the pathway results were interpreted as exploratory annotations that may inform future mechanistic investigations rather than as direct evidence of changes in host intracellular signaling.

### 3.6. Correlation Analysis

**Figure 4:**
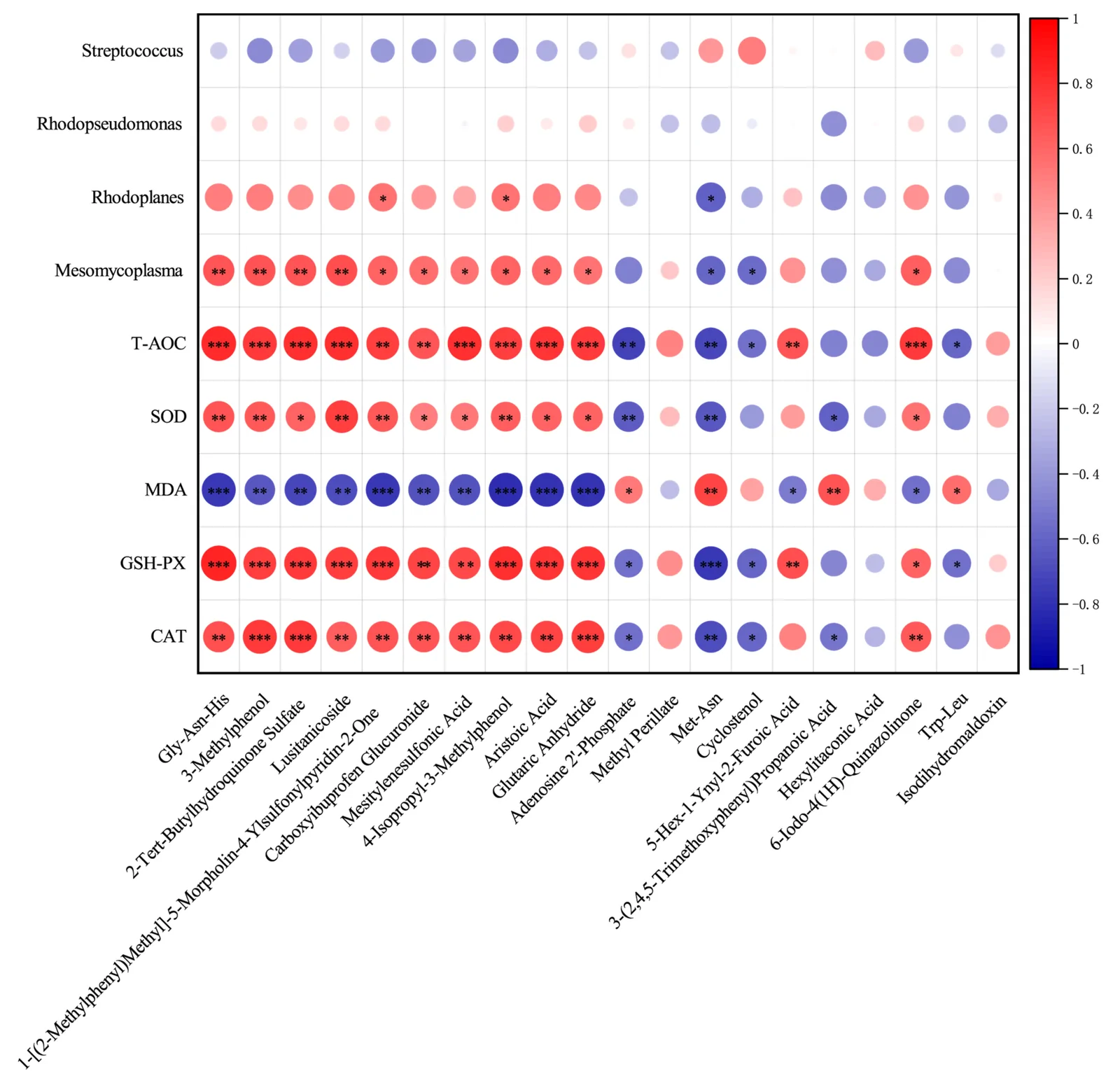
Spearman correlation analysis among differential bacterial genera, the top 20 differential metabolites ranked by VIP value, and antioxidant parameters. Red indicates positive correlations, whereas blue indicates negative correlations. The size of each circle represents the absolute value of the Spearman correlation coefficient. * *q* < 0.05, ** *q* < 0.01, and *** *q* < 0.001.

To explore potential statistical associations among selected fecal bacterial genera, metabolite features, and serum antioxidant parameters, Spearman correlation analysis was performed using four candidate discriminatory genera selected through exploratory LEfSe analysis, the top 20 metabolite features ranked by VIP scores, and five serum antioxidant parameters (Figure 4) (Table S6). Benjamini–Hochberg false discovery rate (FDR) correction was applied across the 180 correlation tests, of which 93 remained statistically significant (q < 0.05). These findings were interpreted as exploratory associations rather than evidence of functional or causal relationships.

Regarding the correlations between bacterial genera and differential metabolites, no significant correlations were observed between Streptococcus or Rhodopseudomonas and the top 20 differential metabolites ranked by VIP after FDR correction. The differential genus Rhodoplanes was significantly positively correlated with 4-Isopropyl-3-Methylphenol and 1-[(2-Methylphenyl)Methyl]-5-Morpholin-4-Ylsulfonylpyridin-2-One (*q* < 0.05), but significantly negatively correlated with Met-Asn (*q* < 0.05). The differential genus Mesomycoplasma was significantly positively correlated with Gly-Asn-His, 3-Methylphenol, 2-Tert-Butylhydroquinone Sulfate, Lusitanicoside, 1-[(2-Methylphenyl)Methyl]-5-Morpholin-4-Ylsulfonylpyridin-2-One, Carboxyibuprofen Glucuronide, Mesitylenesulfonic Acid, 4-Isopropyl-3-Methylphenol, Aristoic Acid, Glutaric Anhydride, and 6-Iodo-4(1H)-Quinazolinone (*q* < 0.05), but significantly negatively correlated with Met-Asn and Cyclostenol (*q* < 0.05).

In the correlation analysis between antioxidant parameters and differential metabolites, T-AOC was significantly positively correlated with Gly-Asn-His, 3-Methylphenol, 2-Tert-Butylhydroquinone Sulfate, Lusitanicoside, 1-[(2-Methylphenyl)Methyl]-5-Morpholin-4-Ylsulfonylpyridin-2-One, Carboxyibuprofen Glucuronide, Mesitylenesulfonic Acid, 4-Isopropyl-3-Methylphenol, Aristoic Acid, Glutaric Anhydride, 5-Hex-1-Ynyl-2-Furoic Acid, and 6-Iodo-4(1H)-Quinazolinone (q < 0.05), but significantly negatively correlated with Adenosine 2′-Phosphate, Met-Asn, Cyclostenol, and Trp-Leu (q < 0.05). SOD was significantly positively correlated with Gly-Asn-His, 3-Methylphenol, 2-Tert-Butylhydroquinone Sulfate, Lusitanicoside, 1-[(2-Methylphenyl)Methyl]-5-Morpholin-4-Ylsulfonylpyridin-2-One, Carboxyibuprofen Glucuronide, Mesitylenesulfonic Acid, 4-Isopropyl-3-Methylphenol, Aristoic Acid, Glutaric Anhydride, and 6-Iodo-4(1H)-Quinazolinone (q < 0.05), but significantly negatively correlated with Adenosine 2′-Phosphate, Met-Asn, and 3-(2,4,5-Trimethoxyphenyl)Propanoic Acid (q < 0.05). MDA was significantly positively correlated with Adenosine 2′-Phosphate, Met-Asn, 3-(2,4,5-Trimethoxyphenyl)Propanoic Acid, and Trp-Leu (q < 0.05), but significantly negatively correlated with Gly-Asn-His, 3-Methylphenol, 2-Tert-Butylhydroquinone Sulfate, Lusitanicoside, 1-[(2-Methylphenyl)Methyl]-5-Morpholin-4-Ylsulfonylpyridin-2-One, Carboxyibuprofen Glucuronide, Mesitylenesulfonic Acid, 4-Isopropyl-3-Methylphenol, Aristoic Acid, Glutaric Anhydride, 5-Hex-1-Ynyl-2-Furoic Acid, and 6-Iodo-4(1H)-Quinazolinone (q < 0.05). GSH-PX was significantly positively correlated with Gly-Asn-His, 3-Methylphenol, 2-Tert-Butylhydroquinone Sulfate, Lusitanicoside, 1-[(2-Methylphenyl)Methyl]-5-Morpholin-4-Ylsulfonylpyridin-2-One, Carboxyibuprofen Glucuronide, Mesitylenesulfonic Acid, 4-Isopropyl-3-Methylphenol, Aristoic Acid, Glutaric Anhydride, 5-Hex-1-Ynyl-2-Furoic Acid, and 6-Iodo-4(1H)-Quinazolinone (q < 0.05), but significantly negatively correlated with Adenosine 2′-Phosphate, Met-Asn, Cyclostenol, and Trp-Leu (q < 0.05). CAT was significantly positively correlated with Gly-Asn-His, 3-Methylphenol, 2-Tert-Butylhydroquinone Sulfate, Lusitanicoside, 1-[(2-Methylphenyl)Methyl]-5-Morpholin-4-Ylsulfonylpyridin-2-One, Carboxyibuprofen Glucuronide, Mesitylenesulfonic Acid, 4-Isopropyl-3-Methylphenol, Aristoic Acid, Glutaric Anhydride, and 6-Iodo-4(1H)-Quinazolinone (q < 0.05), but significantly negatively correlated with Adenosine 2′-Phosphate, Met-Asn, Cyclostenol, and 3-(2,4,5-Trimethoxyphenyl)Propanoic Acid (q < 0.05). Collectively, these results revealed statistical associations among selected fecal bacterial genera, metabolite features, and antioxidant parameters in the CON and 3%Thy groups. However, these correlations do not establish causal relationships or demonstrate that microbial or metabolic changes mediated the antioxidant responses to thymol supplementation.

## 4. Discussion

### 4.1. Effects of Thymol Supplementation in Health Care Sand on Growth Performance and Nutrient Utilization in Squabs

Growth performance is one of the most direct indicators for evaluating the effectiveness of nutritional regulation. In the present study, thymol supplementation in health care sand did not exert significant effects on the growth performance of squabs after Benjamini–Hochberg false discovery rate (FDR) correction within the corresponding biological outcome domain. All replicates were supplied with the same daily feed allowance, and routine visual observations revealed no obvious refusals. Nevertheless, because feed refusals and potential spillage were not quantitatively measured, actual feed intake could not be determined with sufficient precision to exclude small differences among treatments. Therefore, the relationship between thymol supplementation, feed intake, and growth performance could not be fully established under the present experimental conditions.

Beyond statistical significance, the magnitude and potential practical relevance of the observed growth responses should also be considered. The mean total weight gains over the 30-day experimental period were 45.43, 48.78, 53.83, and 50.55 g per squab in the CON, 2%Thy, 3%Thy, and 4%Thy groups, respectively. Compared with the CON group, these values corresponded to numerical increases of approximately 7.4%, 18.5%, and 11.3% in the three thymol-supplemented groups. Although the 3%Thy group showed the largest numerical increase in weight gain, its mean final body weight was only 8.18 g higher than that of the CON group. Furthermore, none of the growth performance outcomes remained statistically significant after FDR correction. Therefore, these numerical differences should not be considered established improvements in growth performance. Their practical and commercial relevance also remains uncertain because actual feed intake, FCR, production costs, and economic returns were not quantitatively evaluated. Further studies are needed to determine whether such growth responses are reproducible and economically meaningful under commercial production conditions.

Thymol is a plant-derived bioactive compound with biological properties similar to those of monoterpenoid phenols such as carvacrol. Previous studies have suggested that these compounds may influence animal production performance by modulating digestive function, redox homeostasis, and the intestinal microbiota, contributing to the increasing interest in plant-derived feed additives [18]. In poultry, combined supplementation with thymol and carvacrol has been reported to increase final body weight, average daily gain, and feed efficiency while affecting nutrient retention and the cecal microbiota [19]. Hashemipour et al. reported that dietary supplementation with thymol and carvacrol improved body weight gain and feed utilization efficiency in broilers and enhanced digestive enzyme activities in the intestine and pancreas [20]. In addition, pure thymol nanoemulsion has been reported to promote growth, improve gastrointestinal barrier function, and modulate the intestinal microbiota in broilers [21]. The differences between these reports and the present findings may be associated with variations in animal species, supplementation levels, formulations, delivery routes, developmental stages, dietary composition, and husbandry conditions. These factors should be considered when interpreting the effects of thymol supplementation across different poultry production systems.

Similarly, the analysis of apparent nutrient retention and energy metabolizability did not reveal significant treatment effects after FDR correction within the corresponding outcome domain. Accordingly, the present results do not provide sufficient statistical evidence that thymol supplementation improved nutrient utilization or energy metabolism in squabs under the conditions examined. Because the present study employed total excreta collection, the resulting nutrient balance estimates represent apparent retention and energy metabolizability rather than direct measurements of intestinal nutrient digestibility. In birds, urinary and fecal components are excreted together, and urinary nitrogen contributes to the nitrogen content of total excreta. Therefore, the crude protein retention estimates should not be interpreted as direct evidence of enhanced intestinal protein digestion. Previous studies have reported that plant-derived additives containing thymol and carvacrol can improve nutrient digestibility in broilers, potentially through changes in digestive enzyme activity, intestinal mucosal morphology, and the gastrointestinal environment [20]. However, intestinal villus morphology and pancreatic or intestinal digestive enzyme activities were not measured in the present study. Therefore, these proposed mechanisms cannot be directly evaluated using the current data. Furthermore, the findings do not establish a clear dose–response relationship or provide sufficient evidence to identify an optimal thymol supplementation level based on growth performance and nutrient utilization. Further studies incorporating quantitative feed intake measurements, a broader range of supplementation levels, and direct assessments of digestive function are warranted to clarify the effects of thymol supplementation on growth and nutrient utilization in squabs.

Although the herbal preparations and functional supplements were maintained at the same inclusion levels across treatments, increasing thymol supplementation required reductions in medium sand, red clay, and limestone powder. Therefore, the observed treatment effects cannot be attributed exclusively to thymol, as potential effects of these compositional changes cannot be excluded. In addition, the active constituents of some commercial preparations were not fully characterized. Future studies should use better-characterized ingredients and more consistent non-thymol formulations to clarify the specific effects of thymol.

### 4.2. Effects of Thymol Supplementation in Health Care Sand on Serum Biochemical Parameters and Antioxidant Status

In the present study, thymol supplementation in health care sand did not significantly affect serum TC, TG, TP, ALB, GLB, Ua, GLU, or ALP in squabs after correction for multiple testing, whereas ACP was the only serum biochemical parameter that retained a significant overall treatment effect. These findings suggest that most of the measured serum biochemical parameters remained relatively stable across the supplementation levels examined. Similarly, Li et al. [22] reported that different levels of thymol–carvacrol cocrystal supplementation had no significant effects on several basic serum biochemical parameters, including TP, ALB, GLB, and uric acid, in broilers. Nevertheless, the absence of significant changes in these parameters does not necessarily indicate that thymol had no effects on protein, lipid, or glucose metabolism, as serum biochemical measurements provide only a limited assessment of metabolic function. In the present study, ACP activity was lower in all thymol-treated groups than in the CON group, with the lowest activity observed in the 3%Thy group. However, ACP is a phosphohydrolase widely distributed in lysosomes and various tissues, and its serum activity lacks strict organ specificity. Therefore, the decrease in ACP alone cannot be directly interpreted as evidence of improved liver, skeletal, or other specific tissue function. This finding may reflect changes in cellular metabolism or tissue turnover, although its physiological significance remains unclear and requires further investigation using organ-specific functional indicators.

Compared with the serum biochemical parameters, the antioxidant indices showed a more consistent response to thymol supplementation in health care sand. After correction for multiple testing, all five antioxidant parameters, including T-AOC, SOD, GSH-PX, CAT, and MDA, retained significant overall treatment effects. Thymol supplementation was associated with increased antioxidant enzyme activities and total antioxidant capacity, together with reduced MDA levels, indicating changes in the antioxidant status of squabs. These findings are generally consistent with previous studies in poultry. Hashemipour et al. [20] reported that thymol and carvacrol increased SOD and GSH-PX activities in the serum and liver of broilers while reducing MDA levels. Placha et al. [23] also observed modulation of the antioxidant defense system in broilers continuously fed thyme essential oil containing thymol. From a mechanistic perspective, the phenolic hydroxyl group in the thymol molecule can participate in free-radical scavenging, while the antioxidant effects of plant-derived bioactive compounds may also involve modulation of endogenous cellular protective pathways. Mountzouris et al. [24] reported that plant-derived additives based on herbs such as thyme upregulated Nrf2 and several cytoprotective genes, including GPX, SOD, and NQO1, and increased total antioxidant capacity in the intestine of broilers. Furthermore, Yang et al. [11] reported that a thymol–carvacrol cocrystal modulated the expression of Keap1/Nrf2-related genes in the liver and intestine of broilers and enhanced antioxidant capacity associated with SOD, CAT, and glutathione. Collectively, these findings suggest that the changes in antioxidant parameters observed in the present study may be related to both the direct antioxidant properties of thymol and its potential influence on endogenous antioxidant defense systems. However, because Nrf2/Keap1 signaling and antioxidant enzyme gene expression were not measured in the present experiment, these mechanisms remain speculative and require further investigation.

### 4.3. Effects of Thymol Supplementation in Health Care Sand on the Fecal Microbiota of Squabs

The intestinal microbiota is a major component of the complex intestinal environment. The intestine harbors a highly diverse and functionally complex microbial community that, together with the intestinal contents and tissues, forms a dynamic and relatively stable ecosystem. These components collectively regulate the digestion and absorption of nutrients and play important roles in maintaining intestinal health and supporting animal growth and development. In the present study, no significant differences were observed in the measured α-diversity indices between the CON and 3%Thy groups. PCA and unweighted UniFrac-based PCoA showed overlapping sample distributions, and PERMANOVA did not detect a statistically significant difference in overall fecal microbial community composition between the two groups. These findings suggest that the present analyses did not provide sufficient statistical evidence of broad changes in fecal microbial diversity or overall community composition following thymol supplementation. Nevertheless, the absence of a statistically significant difference does not exclude more subtle changes in specific microbial taxa or community functions. Given the limited sample size and the exploratory nature of the microbiome analysis, these findings should be interpreted cautiously. In addition, conventional comparisons of relative abundance at both the phylum and genus levels did not reach statistical significance after FDR correction. Taken together, the present analyses did not detect statistically significant differences in overall fecal microbial diversity or community composition between the CON and 3%Thy groups. Nevertheless, the absence of statistical significance does not demonstrate equivalence between the groups or exclude smaller changes in particular microbial populations. However, this does not necessarily indicate that thymol had no effect on microbial function in the intestine, because functional changes in the microbiota do not always require pronounced alterations in community richness or overall structure. Studies of plant-derived essential oils have similarly shown that phytogenic additives can affect specific bacterial populations and their metabolic outputs without causing broad ecological disruption [18].

Although conventional genus-level abundance comparisons did not reveal statistically significant differences after FDR correction, exploratory LEfSe analysis highlighted Streptococcus in the CON group and Rhodopseudomonas, Rhodoplanes, and Mesomycoplasma in the 3%Thy group as candidate discriminatory genera. The biological relevance of these exploratory taxa remains uncertain and requires independent validation. In addition, a single bacterial genus may contain species with markedly different functions or even opposite ecological roles. Therefore, 16S rRNA gene-based genus-level annotation alone is insufficient to classify Streptococcus simply as a “harmful bacterium” or to define Rhodopseudomonas, Rhodoplanes, or Mesomycoplasma directly as “beneficial bacteria.” Their physiological significance requires further validation through species-level identification, metagenomic analysis, or isolation and culture. Although the functional profiles predicted by Tax4Fun2 showed between-group trends in several pathways, none reached statistical significance after FDR correction. Therefore, these predicted results should not be directly interpreted as evidence that thymol altered functions such as carbohydrate, lipid, or amino acid metabolism. Taken together, no statistically significant differences in overall fecal microbial diversity or community composition were detected between the CON and 3%Thy groups. The genera identified by LEfSe may represent candidate taxonomic differences that warrant further investigation, but these findings do not establish selective microbial modulation by thymol.

An additional methodological limitation concerns the exclusion of samples from the fecal microbiome analysis. Following the post hoc exclusion of one CON sample with unusually high richness estimates, one otherwise valid sample from the 3%Thy group was randomly removed solely to maintain equal group sizes. However, equal sample sizes were not required for the statistical comparisons used in this study. The exclusion of this additional sample reduced the available information and may have influenced the stability of the exploratory findings. Because a sensitivity analysis including all technically valid samples was not performed, the robustness of the microbiome results to these exclusion decisions remains uncertain. Therefore, the taxonomic and diversity results should be interpreted with this additional limitation in mind.

### 4.4. Effects of Thymol on the Fecal Metabolome of Squabs

Untargeted metabolomics can provide a global view of changes in metabolic end products and intermediates following nutritional intervention and therefore offers relatively comprehensive information for evaluating the effects of thymol on the intestinal metabolic environment of squabs. In the present study, exploratory untargeted metabolomic analysis revealed differences in fecal metabolite profiles between the CON and 3%Thy groups. PCA revealed a certain degree of separation between samples from the CON and 3%Thy groups, although partial overlap remained, whereas supervised PLS-DA showed relatively clear separation between the two groups. These observations suggest differences in fecal metabolic profiles between the CON and 3%Thy groups despite the absence of a statistically significant difference in overall fecal microbial community composition. However, the exploratory two-group design does not establish the causal mechanisms underlying these metabolic differences. Venn analysis showed that, among the 2201 metabolites detected in the CON and 3%Thy groups, 2032 were shared between the two groups, whereas 105 and 64 metabolites were unique to the CON and 3%Thy groups, respectively. The fact that the vast majority of metabolites were shared between the two groups indicates that the core fecal metabolite composition of squabs remained highly stable following supplementation of health care sand with 3% thymol, although changes occurred in a relatively small subset of metabolites. Further differential metabolite analysis identified a total of 17 differential metabolites, of which 15 showed increased abundance, and 2 showed decreased abundance. Among the 17 differential fecal metabolite features identified in the exploratory comparison between the CON and 3%Thy groups, 15 showed higher relative abundances, and two showed lower relative abundances in the 3%Thy group. These observations do not establish a dose-dependent metabolic response or a confirmed thymol-mediated mechanism. According to the Superclass classification (Table S7), the 17 differential metabolites that remained statistically significant after Benjamini–Hochberg false discovery rate (BH-FDR) correction were assigned to seven categories. Benzenoids represented the largest category, comprising five metabolites (29.41%), followed by Organoheterocyclic compounds, comprising three metabolites (17.65%). Lipids and lipid-like molecules, Organic acids and derivatives, Organic oxygen compounds, and Unknown each contained two metabolites (11.76%), whereas Organic nitrogen compounds contained one metabolite (5.88%). Among these 17 differential metabolites, 15 showed increased abundance, and 2 showed decreased abundance in the 3%Thy group. The differential fecal metabolite features observed in the exploratory CON versus 3%Thy comparison were mainly assigned to putative chemical classes, including benzenoids, organoheterocyclic compounds, lipids and lipid-like molecules, organic acids and derivatives, and organic oxygen compounds. Alterations in benzenoids represented one of the more prominent metabolic features observed in the present study. The benzenoid-related annotations included 3-Methylphenol, Mesitylenesulfonic Acid, 4-Isopropyl-3-Methylphenol, and putatively annotated monobutyl phthalate isomers, whose specific structures could not be reliably distinguished using the available MS/MS evidence. Among these, 3-Methylphenol and 4-Isopropyl-3-Methylphenol had relatively high VIP values, indicating that they contributed substantially to the differences in metabolic profiles between the CON and 3%Thy groups. Thymol itself is a monoterpenoid phenolic compound and therefore has a clear biochemical basis for absorption and biotransformation following ingestion. Pisarčíková et al. [25] detected thymol sulfate and thymol glucuronide in the plasma, liver, and duodenal wall of broilers, demonstrating that poultry can metabolize thymol through conjugation reactions such as sulfation and glucuronidation. Oceľová et al. [26] further reported that, following continuous intake of thyme essential oil containing thymol, thymol could be detected in the contents of the duodenum, jejunum, ileum, cecum, and colon of broilers, suggesting that the gastrointestinal tract is an important site for thymol absorption and biotransformation. Therefore, the changes in aromatic and phenolic candidate metabolites observed in the present study may be associated with the transformation processes occurring after exogenous thymol enters the digestive tract. However, there is currently insufficient evidence to demonstrate that specific differential metabolites, such as 3-Methylphenol and 4-Isopropyl-3-Methylphenol, are directly derived from thymol biotransformation. Therefore, a direct precursor–product relationship between thymol and these metabolites should not be inferred from the present results.

In addition to aromatic compounds, small peptides and nitrogen-containing compounds also showed notable responses. In the present study, the abundance of Gly-Asn-His was increased in the 3%Thy group, whereas that of Met-Asn was decreased. In addition, the top 20 differential metabolites ranked by VIP included Gly-Asn-His, Met-Asn, and Trp-Leu. KEGG annotation also involved amino acid-related compounds such as pipecolic acid and carnosine and pointed to processes including lysine degradation, histidine metabolism, and β-alanine metabolism. These findings suggest that thymol may influence the composition of amino acids, small peptides, and their degradation products in feces, thereby altering the metabolic characteristics of nitrogen-containing compounds. Previous studies have shown that thymol intervention can affect arginine and proline metabolism, arginine biosynthesis, and other amino acid-related metabolic pathways [27]. Because the animal model used in that study differed from that of the present study, those findings mainly support the possibility that thymol can modulate amino acid metabolism and cannot be regarded as direct evidence for specific metabolic mechanisms in squabs.

Lipids and lipid-like molecules were also an important metabolic category of interest in the present study. Based on the overall untargeted metabolomics profile, lipids and lipid-like molecules represented the largest category among metabolites with Superclass classification information, comprising 520 metabolites and accounting for 38.63% of the total. This finding indicates that lipid-related molecules constitute an important component of the basal fecal metabolic profile of squabs. Among the differential metabolites, aristoic acid and methyl perillate were both classified as lipids and lipid-like molecules, showed increased abundance in the 3%Thy group, and had relatively high VIP values. In addition, KEGG enrichment analysis involved lipid-related processes such as fatty acid degradation and the PPAR signaling pathway, suggesting that thymol treatment may be accompanied by alterations in the metabolic environment of fatty acids and lipid derivatives. This interpretation is biologically plausible. Hashemipour et al. [20] reported that dietary supplementation with thymol and carvacrol altered serum and muscle fatty acid composition in broilers, increased the proportion of polyunsaturated fatty acids, increased SOD and GSH-Px activities, and reduced MDA levels, indicating that monoterpenoid phenolic phytochemicals can simultaneously influence lipid composition and lipid oxidative status [20]. In addition, an untargeted metabolomics study by Ma et al. [27] found that thymol intervention was accompanied by changes in lipid-related processes such as glycerophospholipid metabolism, further supporting a potential association between thymol and lipid metabolic networks. Notably, EE digestibility and serum TC and TG were not significantly altered in the 3%Thy group in the present study. Therefore, the changes in fecal lipid-related metabolites cannot simply be interpreted as evidence of enhanced fat digestibility or increased systemic lipid metabolism. A more cautious interpretation is that thymol may selectively influence the composition and relative abundance of specific lipids and lipid derivatives in the distal digestive tract without markedly altering overall fat digestion or circulating lipid levels. PPARα is an important transcriptional regulator of fatty acid uptake, mitochondrial and peroxisomal fatty acid oxidation, and overall lipid homeostasis. Although some differential metabolites in the present study were mapped to PPAR-related pathways [28], the current findings were derived from KEGG mapping of fecal metabolites, and tissue PPAR expression or activity was not measured. Therefore, these results can only indicate that the relevant metabolites are associated with lipid metabolism and PPAR-related metabolic networks and should not be interpreted as evidence that thymol activates or inhibits the PPAR signaling pathway. The same limitation applies to the KEGG associations involving MAPK, insulin, and cGMP-PKG signaling pathways. Because the present study analyzed fecal metabolites rather than host tissue signaling activity, these annotations cannot establish changes in intracellular pathway activation or inhibition. Verification of the potential involvement of these pathways would require direct tissue-level molecular analyses.

Organoheterocyclic compounds also constituted part of the metabolic response observed in the present study. The abundances of 1-[(2-Methylphenyl)Methyl]-5-Morpholin-4-Ylsulfonylpyridin-2-One, Glutaric Anhydride, and 5-Hex-1-Ynyl-2-Furoic Acid were increased in the 3%Thy group, with the first two metabolites exhibiting relatively high VIP values. These findings suggest that thymol treatment may influence the composition and relative abundance of certain heterocyclic small molecules in the feces of squabs. However, because heterocyclic compounds differ substantially in chemical structure and metabolic origin, and because there is currently no direct evidence demonstrating a clear causal relationship between the observed changes in these organoheterocyclic compounds and thymol metabolism or regulation, the present findings are more appropriately interpreted as part of the selective remodeling of the fecal metabolic environment following thymol treatment. They should not be taken as evidence that thymol directly promotes the formation of these organoheterocyclic compounds. The specific origins of these compounds and their biological significance in the response to thymol require further investigation.

Taken together, the exploratory comparison between the CON and 3%Thy groups identified differences in 17 fecal metabolite features, including compounds putatively associated with aromatic molecules, amino acids and small peptides, and lipids. KEGG annotation suggested database-derived associations with selected amino acid- and lipid-related pathways. However, these observations do not establish a dose-dependent metabolic response or demonstrate that the observed differences were specific to the 3%Thy level, because the 2%Thy and 4%Thy groups were not included in the metabolomic analysis. Furthermore, the identities and biological origins of some metabolite features remain uncertain. Therefore, the present metabolomic findings should be regarded as preliminary evidence for further investigation rather than as confirmation of a specific thymol-induced metabolic mechanism or an optimal supplementation level.

It should be emphasized that the fecal microbiome and metabolomic analyses were exploratory comparisons restricted to the CON and 3%Thy groups. The 3%Thy group was selected after evaluation of the phenotypic and physiological responses rather than being prespecified before the experiment. Therefore, these omics findings should not be interpreted as independent evidence of a dose-dependent thymol response or as confirmation that 3% represents an optimal supplementation level. Moreover, because the 2%Thy and 4%Thy groups were not included in the omics analyses, it remains unknown whether the observed microbial and metabolic differences were specific to the 3%Thy treatment or were shared across thymol-supplemented groups.

### 4.5. Associations Among Differential Bacterial Genera, Differential Metabolites, and Antioxidant Status

The correlation analyses identified exploratory statistical associations among LEfSe-selected candidate bacterial genera, putatively annotated fecal metabolite features, and serum antioxidant parameters. Because the conventional taxonomic abundance comparisons did not reveal significant differences after FDR correction and the chemical identities of some metabolite features remain uncertain, these associations should be interpreted cautiously. They do not establish treatment-induced microbial changes, functional interactions, mediation, or causal relationships.

Among the associations between bacterial genera and metabolites, *Rhodoplanes* and *Mesomycoplasma* exhibited relatively distinct metabolite-associated patterns. Specifically, *Rhodoplanes* was significantly associated with 4-Isopropyl-3-Methylphenol, whereas *Mesomycoplasma* showed significant associations with several metabolites with relatively high VIP values, including Gly-Asn-His, 3-Methylphenol, 2-Tert-Butylhydroquinone Sulfate, Lusitanicoside, and 4-Isopropyl-3-Methylphenol, while showing associations in the opposite direction with Met-Asn and Cyclostenol. These findings suggest that alterations in specific bacterial genera in the feces of thymol-treated squabs may occur concurrently with changes in aromatic compounds, small peptides, organic acids, and lipid-related metabolites. Previous studies have shown that thymol intervention can simultaneously alter the intestinal microbial composition and amino acid- and lipid-related metabolic networks, suggesting that the effects of thymol may involve coordinated regulation of the intestinal microecology and metabolic phenotype [26]. Among these metabolites, aromatic candidate compounds such as 3-Methylphenol and 4-Isopropyl-3-Methylphenol also showed strong associations with certain differential bacterial genera and therefore warrant further investigation. Thymol itself is a monoterpenoid phenolic compound and can undergo further biotransformation in poultry through processes such as sulfation and glucuronidation. Therefore, changes in aromatic metabolites may reflect alterations in the chemical environment of the digestive tract and related metabolic processes following thymol intake. Studies in broilers have detected thymol sulfate and thymol glucuronide in plasma, liver, and duodenal wall tissues, demonstrating that poultry can undergo substantial phase II metabolism of thymol [27]. However, because isotope tracing and functional validation of bacterial strains were not performed in the present study, these aromatic metabolites can currently only be considered statistically associated with thymol treatment and changes in specific bacterial genera. It cannot yet be confirmed that they are thymol metabolites directly produced by particular bacterial genera. In addition to the associations between bacterial genera and metabolites, the relationships between metabolites and antioxidant parameters showed a more consistent pattern. Several metabolites with relatively high VIP values, including Gly-Asn-His, 3-Methylphenol, and 2-Tert-Butylhydroquinone Sulfate, were generally significantly positively correlated with T-AOC, SOD, GSH-Px, and CAT, but significantly negatively correlated with MDA, an indicator of lipid peroxidation. In contrast, Met-Asn generally showed the opposite correlation pattern. The consistent directions of association across multiple antioxidant parameters suggest that some thymol-induced metabolic changes may occur in parallel with improvements in antioxidant capacity. However, because the activities or expression of related antioxidant genes were not examined in the present study, these metabolites are more appropriately regarded as metabolic biomarkers reflecting changes in antioxidant status following thymol intervention rather than as evidence that the metabolites themselves directly improve antioxidant status.

Existing poultry studies have shown that monoterpenoid phenolic compounds such as thymol and carvacrol can increase the activities of antioxidant enzymes, including SOD and GSH-PX, while reducing lipid peroxidation, indicating that thymol has a clear potential to modulate redox homeostasis in poultry [20]. In addition, phytogenic feed additives have been reported to enhance intestinal antioxidant capacity in broilers and regulate the expression of cytoprotective genes such as Nrf2, GPX, SOD, and NQO1, further suggesting that metabolic changes induced by plant-derived bioactive compounds may contribute to the regulation of antioxidant capacity in animals [24]. Overall, the correlation analyses in the present study showed relatively stable associations between certain differential bacterial genera and specific differential metabolites following thymol treatment, while these metabolites also exhibited consistent directions of correlation with host antioxidant status. These findings revealed an exploratory pattern of associations among selected fecal bacterial genera, metabolite features, and host antioxidant parameters. Although these associations may help generate hypotheses regarding the biological responses to thymol supplementation, they do not demonstrate that microbial or metabolic alterations mediate changes in host antioxidant status. Among these, *Rhodoplanes*, *Mesomycoplasma*, and their associated aromatic, small peptide, lipid-related, and organic acid metabolites may represent candidate nodes worthy of further investigation. However, the present study cannot demonstrate that a specific bacterial genus directly produces or degrades the associated metabolites, nor can it establish that a specific metabolite directly regulates host antioxidant capacity. Therefore, the correlation results of the present study should still be regarded as exploratory evidence. Future studies should employ targeted metabolomics, bacterial strain culture or colonization experiments, metabolite supplementation experiments, and tissue-level molecular validation to further clarify the specific roles of key bacterial genera and metabolites in the regulation of physiological status in squabs by thymol.

## 5. Conclusions

Under the conditions of the present study, thymol supplementation in health care sand was associated with favorable changes in serum antioxidant parameters in squabs, including increased antioxidant enzyme activities and reduced malondialdehyde levels. Although the 3%Thy group showed the highest numerical total weight gain among the treatments evaluated, growth performance, apparent nutrient retention, and energy metabolizability did not differ significantly among groups after false discovery rate correction. Exploratory fecal microbiome and metabolomic analyses were conducted only between the CON and the retrospectively selected 3%Thy groups. No statistically significant difference in overall fecal microbial community composition was detected, whereas differences were observed in 17 candidate fecal metabolite features. These omics findings cannot establish a dose-dependent response or demonstrate that the 3%Thy treatment was superior to the other supplementation levels. Overall, 3%Thy may be considered a comparatively suitable candidate supplementation level for further investigation among those examined, but the present study does not establish an optimal thymol inclusion level.

The present study has several limitations. First, the fecal microbiome and metabolomic analyses were restricted to the CON and 3%Thy groups, and the 3%Thy group was selected after evaluation of the phenotypic and physiological responses rather than being prespecified before the experiment. Therefore, these omics analyses were exploratory and cannot establish a dose-dependent response or determine whether the observed microbial and metabolic changes were specific to the 3%Thy treatment. Second, 16S rRNA gene sequencing and Tax4Fun2 were used to characterize microbial community composition and predict microbial functions; therefore, the functional information remains inferential and cannot accurately resolve species-level microbial changes or directly reflect actual microbial metabolic functions. In addition, the post hoc exclusion of one CON microbiome sample and the subsequent random exclusion of one 3%Thy sample to balance group sizes represent methodological limitations that may affect the robustness of the exploratory microbiome findings. Third, although significant correlations were identified among certain bacterial genera, differential metabolites, and antioxidant parameters, these associations do not establish functional interactions or causal relationships. Fourth, although the same daily feed allowance was supplied to each replicate and no obvious refusals were observed, feed refusals and potential spillage were not quantitatively weighed. Therefore, actual feed intake could not be accurately determined, FCR could not be reliably calculated, and differences in feed intake among treatments cannot be excluded.

Future studies incorporating all thymol supplementation levels and integrating quantitative feed-intake measurements, metagenomics, targeted metabolomics, tissue-level molecular analyses, and functional validation experiments are warranted to further characterize dose-related responses and clarify the biological mechanisms associated with thymol supplementation in squabs.

## Figures and Tables

**Table 1:** Composition and nutrient levels of the basal diet for squabs (air-dry basis).

Ingredient Composition	Content (%)	Nutrient Composition ^2^	Content
Corn	51.91	Dry matter (%)	88.93
Soybean meal	30.00	Organic matter (%)	85.71
Wheat bran	5.24	Crude protein (%)	14.56
Limestone powder	1.90	Crude fat (%)	5.16
Salt	0.95	Gross energy (MJ/kg)	18.61
Yeast powder	4.76		
Soybean oil	0.95		
Premix ^1^	4.29		
Total	100.00		

^1^ The premix provided the following per kilogram of diet: vitamin A, 27,000 IU; vitamin D_3_, 90,000 IU; vitamin E, 850 mg; vitamin K_3_, 92 mg; vitamin B_2_, 160 mg; vitamin B_6_, 115 mg; niacinamide, 1050 mg; D-calcium pantothenate, 520 mg; D-biotin, 2 mg; folic acid, 20 mg; Cu, 340 mg; Fe, 1700 mg; Mn, 1700 mg; and Zn, 1400 mg. ^2^ The reported nutrient composition values, including gross energy, were determined analytically.

**Table 2:** Composition of health care sand %.

Component	CON	2%Thy	3%Thy	4%Thy
Medium sand	72.30	70.54	69.69	68.81
Red clay	6.00	5.89	5.79	5.74
Limestone powder	4.80	4.67	4.62	4.55
Yeast powder	3.00	3.00	3.00	3.00
Maxing Shigan powder	2.00	2.00	2.00	2.00
Qingfei Zhike powder	2.00	2.00	2.00	2.00
Allicin	2.00	2.00	2.00	2.00
Rouqin Fengzhangtai	2.00	2.00	2.00	2.00
Isatis indigotica root	1.00	1.00	1.00	1.00
Dahuang Soda powder	1.00	1.00	1.00	1.00
Feather nutrient supplement	1.00	1.00	1.00	1.00
Growth-promoting supplement	1.00	1.00	1.00	1.00
Trace elements	1.00	1.00	1.00	1.00
Immunity-enhancing supplement	0.50	0.50	0.50	0.50
Niuchan granules	0.20	0.20	0.20	0.20
Gandan granules	0.20	0.20	0.20	0.20
Thymol	0.00	2.00	3.00	4.00

**Table 3:** Effects of thymol supplementation in health care sand on growth performance and body measurement parameters of squabs. Values within the same row with no superscript letters or with the same superscript letters are not significantly different (*q* > 0.05), whereas values with different superscript letters differ significantly (*q* < 0.05). n = 10 per group.

Item	CON	2%Thy	3%Thy	4%Thy
Body weight g				
Body weight on day 0	502.68 ± 41.70	502.58 ± 32.36	502.45 ± 31.78	502.73 ± 28.99
Body weight on day 30	548.10 ± 38.53	551.35 ± 33.55	556.28 ± 28.60	553.28 ± 26.82
Total weight gain	45.43 ± 6.43	48.78 ± 8.70	53.83 ± 7.26	50.55 ± 7.53
Average daily gain	1.51 ± 0.21	1.63 ± 0.29	1.79 ± 0.24	1.69 ± 0.25
Breast width cm				
Breast width on day 0	5.78 ± 0.24	5.85 ± 0.19	5.92 ± 0.27	5.93 ± 0.19
Breast width on day 30	6.29 ± 0.35	6.41 ± 0.24	6.55 ± 0.29	6.54 ± 0.25
Breast width gain	0.51 ± 0.13	0.56 ± 0.13	0.63 ± 0.08	0.61 ± 0.13
Breast depth cm				
Breast depth on day 0	6.74 ± 0.25	6.72 ± 0.23	6.77 ± 0.24	6.74 ± 0.19
Breast depth on day 30	7.17 ± 0.21	7.17 ± 0.25	7.24 ± 0.25	7.21 ± 0.21
Breast depth gain	0.43 ± 0.07	0.45 ± 0.07	0.47 ± 0.06	0.47 ± 0.09
Shank length cm				
Shank length on day 0	3.27 ± 0.15	3.34 ± 0.09	3.23 ± 0.15	3.27 ± 0.08
Shank length on day 30	3.54 ± 0.14	3.63 ± 0.09	3.54 ± 0.15	3.56 ± 0.08
Shank length gain	0.27 ± 0.05	0.29 ± 0.02	0.31 ± 0.03	0.29 ± 0.02
Keel length cm				
Keel length on day 0	8.73 ± 0.52	8.83 ± 0.39	8.75 ± 0.55	8.76 ± 0.17
Keel length on day 30	9.17 ± 0.52	9.28 ± 0.42	9.23 ± 0.51	9.23 ± 0.18
Keel length gain	0.44 ± 0.10	0.46 ± 0.06	0.48 ± 0.06	0.47 ± 0.05
Oblique body length cm				
Oblique body length on day 0	12.33 ± 0.27	12.48 ± 0.32	12.40 ± 0.29	12.43 ± 0.24
Oblique body length on day 30	12.88 ± 0.31	13.06 ± 0.38	13.02 ± 0.30	13.06 ± 0.24
Oblique body length gain	0.55 ± 0.07	0.58 ± 0.11	0.62 ± 0.09	0.64 ± 0.10
Shank circumference cm				
Shank circumference on day 0	2.18 ± 0.04	2.18 ± 0.04	2.20 ± 0.05	2.19 ± 0.06
Shank circumference on day 30	2.52 ± 0.08	2.54 ± 0.06	2.57 ± 0.07	2.54 ± 0.10
Shank circumference gain	0.34 ± 0.06	0.35 ± 0.05	0.38 ± 0.05	0.35 ± 0.06

**Table 4:** Effects of thymol supplementation in health care sand on apparent nutrient retention and energy metabolizability in squabs. DM, dry matter; OM, organic matter; CP, crude protein; GE, gross energy; EE, ether extract; AME, apparent metabolizable energy. Values within the same row with no superscript letters or with the same superscript letters are not significantly different (*q* > 0.05), whereas values with different superscript letters differ significantly (*q* < 0.05). n = 10 per group.

Item	CON	2%Thy	3%Thy	4%Thy
Apparent DM retention %	66.02 ± 6.29	69.36 ± 4.69	70.64 ± 3.27	69.75 ± 3.74
Apparent OM retention %	66.74 ± 6.07	69.73 ± 4.34	69.98 ± 3.63	70.49 ± 3.4
Apparent CP retention %	38.28 ± 8.99	40.61 ± 13.03	43.47 ± 3.61	40.86 ± 7.48
Apparent GE metabolizability %	70.03 ± 4.69	72.82 ± 4.17	74.01 ± 2.89	73.14 ± 3.44
Apparent EE retention %	68.69 ± 6.78	70.35 ± 8.07	72.62 ± 7.26	71.97 ± 6.83
AME MJ/kg	13.08 ± 0.86	13.66 ± 0.67	13.76 ± 0.54	13.54 ± 0.59

## Data Availability

The raw 16S rRNA gene sequencing data generated in this study have been deposited in the NCBI Sequence Read Archive. The data are publicly available under BioProject accession number PRJNA1531323. The original contributions presented in this study are included in the article/Supplementary Material. Further inquiries can be directed to the corresponding author.

## Supplementary Materials

The following supporting information can be downloaded at https://www.mdpi.com/article/10.3390/ani16203314/s1, Table S1: Data on the effects of thymol supplementation in health care sand on serum biochemical parameters in squabs; Table S2: Data on the effects of thymol supplementation in health care sand on antioxidant parameters in squabs; Table S3: Microbial alpha diversity data; Table S4: Microbial relative abundance data at the phylum level; Table S5: Microbial relative abundance data at the genus level; Table S6: Data on the associations among differential bacterial genera, differential metabolites, and antioxidant status; Table S7: Superclass classification of differential metabolites.

## Abbreviations

The following abbreviations are used in this manuscript:

CON	Control group
Thy	Thymol
DM	Dry matter
GE	Gross energy
ME	Metabolizable energy
EE	Ether extract
MDA	Malondialdehyde
COR	Cortisol
SOD	Superoxide dismutase
GSH-PX	Glutathione peroxidase
CAT	Catalase
ADG	Average daily gain
OM	Organic matter
CP	Crude protein
TC	Total cholesterol
TG	Triglycerides
TP	Total protein
ALB	Albumin
GLB	Globulin
Ua	Uric acid
GLU	Glucose
ACP	Acid phosphatase
ALP	Alkaline phosphatase
T-AOC	Total antioxidant capacity
GPX	Glutathione peroxidase
